# Childhood PFAS exposure and immunotoxicity: a systematic review and meta-analysis of human studies

**DOI:** 10.1186/s13643-024-02596-z

**Published:** 2024-07-09

**Authors:** Evangelia E. Antoniou, Wolfgang Dekant

**Affiliations:** 1 Epicurus-Reviews, MetaAnalyses.Com, Heesstraat 80, Bilzen, Belgium; 2 Department of Toxicology, University of Würzburg, Würzburg, Germany

**Keywords:** Per- and polyfluorinated aliphatic substances, Prenatal, Childhood, Immunomodulation, Humans, PFAS, Meta-analysis, Antibody response, Infections

## Abstract

**Background:**

Exposure to poly- and perfluoroalkyl substances (PFAS) may affect infant and childhood health through immunosuppression. However, the findings of epidemiological literature examining relationships between prenatal/childhood PFAS exposure and vaccine response and infection in humans are still inconclusive. The aim of this review was to examine the effects of PFAS exposure on vaccine antibody response and infection in humans.

**Methods:**

The MEDLINE/Pubmed database was searched for publications until 1 February 2023 to identify human studies on PFAS exposure and human health.

Eligible for inclusion studies had to have an epidemiological study design and must have performed logistic regression analyses of gestational or childhood exposure to PFAS against either antibody levels for pediatric vaccines or the occurrence of children’s infectious diseases. Information on baseline exposure to PFAS (in ng/mL), the age of PFAS exposure (gestational or in years), and the outcome was measured, potentially leading to multiple exposure-outcome comparisons within each study was collected. Percentage change and standard errors of antibody titers and occurrence of infectious diseases per doubling of PFAS exposure were calculated, and a quality assessment of each study was performed.

**Results:**

Seventeen articles were identified matching the inclusion criteria and were included in the meta-analysis. In general, a small decrease in antibody response and some associations between PFAS exposure and childhood infections were observed.

**Conclusions:**

This meta-analysis summarizes the findings of PFAS effects on infant and childhood immune health. The immunosuppression findings for infections yielded suggestive evidence related to PFAS exposure, particularly PFOS, PFOA, PFHxS, and PFNA but moderate to no evidence regarding antibody titer reduction.

**Systematic review registration:**

The research protocol of this systematic review is registered and accessible at the Open Science Framework (https://doi.org/10.17605/OSF.IO/5M2VU).

**Supplementary Information:**

The online version contains supplementary material available at 10.1186/s13643-024-02596-z.

## Background

Since the 1950s, perfluooroctanoate (PFOA; C_7_F_15_ C00^−^) and perfluorooctanatesulfoinate (PFOS; C_8_F_17_ SO_3_^−^), two of several thousands per- and polyfluoroalkyl substances (PFAS), have been widely used for industrial and commercial purposes. PFAS are resistant to environmental degradation, and due to their ability to bioaccumulate into living organisms, they are now present in environmental media and biota, including humans [1].

Exposure to PFOA/PFOS or general PFAS has been suspected to be related to adverse outcomes on children’s health effects such as neurodevelopment, growth, and the immune system [2–4]. Infant and childhood vaccination is intended to offer lasting protection against infectious disease, and an antibody level below protection levels reflects a deficiency in immune function [5].

Immunotoxicity typically has been the focus of regulatory testing for chemical agents due to risks of increased infection. For example, the European Food Safety Authority (EFSA) recently used the association between PFAS exposure with reduced antibody response in humans (children) to derive a tolerable intake for a group of the sum of four PFAS from food [6].

Immunosuppression is the reduced ability of the immune system to respond to a challenge from a level considered normal [7]. The impact of immunosuppression on the general health of an individual can be widely variable from slightly, but still measurable reduced responses to vaccinations to common pathogens as well as the causation of certain cancers [7].

The epidemiological literature examining relationships between PFAS exposure and immunosuppressing effects in humans has been previously reviewed by DeWitt [7], Chang et al. [8], by the National Toxicology Program (NTP) [9], and very recently by von Holst et al. [10]. Common among these reviews is the concern that prenatal and early childhood exposure to PFAS could cause changes in the immune system development.

Chang et al. [8] in a systematic review, which assessed only epidemiologic studies, concluded that the available evidence is insufficient to reach a conclusion about a causal relationship between exposure to PFOS and PFOA and any immune-related health condition in humans. In the most recent review [10], the authors concluded that there was a strong indication of immunosuppression with reduced childhood antibody response to vaccination, particularly with PFOS, PFOA and PFHxS, and some indication of an effect of PFAS exposure on childhood infectious diseases.

NTP [9], on the other hand, concluded that both PFOS and PFOA should be considered an immune hazard to humans; this was based on evidence not only from epidemiologic studies but also from studies in experimental animals and mechanistic studies. Thus, the NTP concluded that evidence for suppression of antibody responses for both PFOA and PFOS was high in experimental animals and moderate in humans. Furthermore, associations of a reduced antibody response in children with PFAS exposures were also applied as endpoints to derive a tolerable exposure limit to PFAS. Other regulatory agencies did not consider such associations as a useful endpoint for human risk characterization due to inconsistencies across studies and/or a smaller number of studies examining these outcomes [11–13]. Nevertheless, no previous review has attempted to synthesize the data via a meta-analysis, where separate groups based on age, exposure, and outcome could be constructed and analyzed.

Therefore, the aim of this review was to combine all the available evidence on the epidemiologic studies of prenatal and child PFAS exposure and vaccine-induced antibody response and to examine the effects of PFAS exposure on vaccine response and common childhood infectious diseases.

## Methods

The results of the meta-analysis were reported according to the 2020 PRISMA guidelines [14].

### Search strategy

We searched MEDLINE/Pubmed for publications until 1 February 2023 to identify all human studies on PFAS exposure and human health using the following the search string: “perfluoro* [tiab] OR pfas[tiab] OR perfluorinated*[tiab] AND human health” without language or other restrictions. Key search terms were required to appear in the article title or abstract. All identified studies were uploaded to Covidence [15] (v2703 7275834a) and automatically screened for duplicates. The title and abstract of the retrieved reports were independently screened by two reviewers as well as the full texts screening for eligibility.

To be eligible for inclusion in our analysis, studies had to have an epidemiological study design, such as cohort, cross-sectional, or case–control studies, and must have performed logistic or multivariate regression analyses with covariate adjustment of gestational or childhood exposure to PFAS against either [1] antibody levels for pediatric vaccines or the occurrence of child’s infectious diseases. Studies were excluded if they presented non-human data, adult data (participants with age >  = 18 years), other immune-related outcomes, asthma or allergies, non-vaccine related antibodies, were reviews or letters, or did not provide suitable data for meta-analyses. This study does not involve human participants.

### Data extraction

Within each study, information was collected on the age that PFAS exposure was measured (gestational or in years) and the age that the outcome was measured, potentially leading to multiple exposure-outcome comparisons within each study. Data extraction was performed by the first author. A second reviewer examined the extracted data for accuracy. These *exposure windows* can be of cross-sectional nature (when exposure and outcome were measured at the same time) or longitudinal (when the outcome was measured at a later time point than the exposure). We assessed the risk of bias for each exposure window by using a tool developed by the Office of Health Assessment and Translation (OHAT) [16].

For each exposure window, we extracted information on baseline exposure to PFOS, PFOA, PFHxS, PFNA, PFDA, PFUnDA, PFDoDA, or PFTrDA (in ng/mL). As levels of PFAS are strongly right skewed, we assumed that these were log2-transformed if not reported otherwise. Within the same exposure windows, we extracted or recalculated the percentage change (%D) with the corresponding standard errors (SE) of the antibody titers for diphtheria, tetanus, measles, mumps, haemophilus influenza type b (Hib) or hand, foot, and mouth disease (EV71 and CA16) per doubling of PFAS exposure. Odds ratios (OR) with the corresponding standard error (SE) were extracted or recalculated for throat infection (including coughing and streptococcus infection), rhinitis (including rhinitis-conjunctivitis), respiratory infection (including upper respiratory infections, pneumonia, and bronchitis), otitis media, gastrointestinal infection (including diarrhea and gastroenteritis), dermatitis (including atopic dermatitis), fever (including flu), hospitalization due to any infection, any infection, cold, pseudocroup, and chickenpox. If multiple ORs were reported by the authors, the OR representing the highest contrast (i.e., highest quartile) between PFAS exposures and antibody response or infection was chosen.

### Data analysis and synthesis

We tabulated the exposure windows for each PFAS and % change in antibody responses for the different vaccines. If more than one exposure windows were available, the results were pooled in a random effects meta-analysis using the r-package *metagen,* and the pooled results were tabulated together with the number of exposure windows that were combined but stratified by gestational vs. childhood exposure. Models fully adjusted for confounding variables, as identified and reported within each study, were used for the pooled analyses.

Estimations of the between-study result heterogeneity were performed using the I-squared (I^2^). I^2^ is assumed to be low (< 25%), moderate (25–75%), or high (> 75%) [17]. This approach was repeated for the ORs regarding PFAS exposures and childhood infections. As it is likely that different exposure windows within each study are dependent, we conducted a multi-level sensitivity analysis, in which first, exposure windows within studies were pooled, and afterwards, the study-level results. This analysis showed no differences (data not shown). All forest plots are available in the supplementary file (Figs. S1–S7).

### Confidence of rating: assessment of body of evidence

The Grading of Recommendations Assessment, Development, and Evaluation system (GRADE) was used to evaluate the quality of evidence for each immune and infectious disease outcome [18]. The studies included in the meta-analysis were grouped by key study design features, and each grouping of studies was given an initial confidence rating by those features. The quality rating began with the study design and afterwards was decided whether to downgrade based on five features (risk of bias, unexplained inconsistency, indirectness or lack of applicability, imprecision, and publication bias), or to upgrade based on three features (large magnitude of effect, dose response, consistency across study designs/populations, and consideration of residual confounding). A risk of bias heatmap, created with the Robvis visualization tool [19], is presented separately in the supplementary table S1. Imprecision was evaluated by *p*-values and CIs, and the number of studies. Indirectness meant discrepancies in populations and measurements of the outcomes among the studies having semblable results and evaluation criteria. In the supplementary tables S2 and S3, an explanation of the GRADE system is provided.

The GRADE scores for the major results from the included studies were summarized in supplementary tables S4–S19.

The confidence ratings were translated into a level of confidence of health effects for each type of health outcome separately according to one of the four statements: (1) High, (2) Moderate, (3) Low, or (4) Very Low.

In the context of identifying research needs, a conclusion of “High confidence” indicates that further research is very unlikely to change confidence in the apparent relationship exposure to PFAS and the outcome. Contrarywise, a conclusion of “Very Low confidence” suggests that further research is very likely to have an impact on confidence in the association between exposure to PFAS and the outcome. Confidence ratings were assessed by two reviewers independently, and disagreement was solved by discussion.

## Results

### Study selection

Of the 3094 studies imported in our database for screening, 514 were removed as duplicates.

**Fig. 1:**
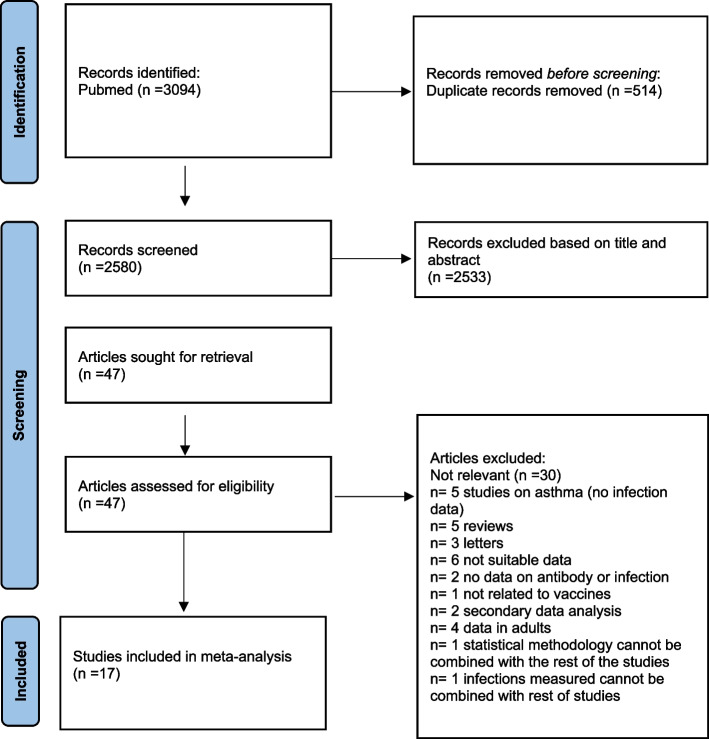
Selection of studies for meta-analyses

Of the remaining 2580 screened studies, 2533 were considered irrelevant based on title and abstract. Of the remaining 47 articles that were assessed in full text, 30 studies were excluded because they reported on outcomes not relevant to this study, or were reviews/letters, or reported on secondary data analyses or the statistical methodology used in the article could not be combined with the rest of the studies. Finally, 17 articles, 15 birth cohort studies, and two cross-sectional studies were identified as relevant and included in the meta-analysis. A flow chart of the study selection can be found in Fig. 1.

The main characteristics of the study population included in the meta-analysis contributing PFAS samples and the outcome measurement (age when the antibody response and/or the infectious diseases measured) are presented in Table 1. Baseline PFAS values are presented in two exposure windows: during gestation (0 years), and during childhood (4 months, 18 months, 7 years, and 13 years). The time (age of the children) of the outcome measurement (effect statistics in table) ranged from 3 months to 14.5 years. The sample contributing data for the analyses ranged from *n* = 49 to *n* = 2689. In addition, three studies analyzed PFAS and outcome measurements cross-sectionally, at 5 years of age [3, 20] and at 7 years of age [21, 22].

**Table 1:** Study characteristics of the population included in meta-analyses

Study *Author (reference)*	Baseline PFAS (ng/ml) *median (IQR) or mean (sd)*		Outcome age *(Sample size)*	Effect statistics^a^
**Ait Bamai** [23] Japan	**Gestation** PFOS 5.1 (3.8–7.0) PFOA 1.9 (1.3–2.9) PFHxS 0.3 (0.2–0.4 PFNA 1.1 (0.9–1.5) PFDA 0.5 (0.4–0.7) PFUnDA 1.4 (1.0–1.9) PFDoDA 0.2 (0.1–0.2) PFTrDA 0.3 (0.2–0.4)		**7 years** (*n* = 2689)	Rhinitis (OR)^b^ Chicken pox (OR) Otitis media (OR) Pneumonia (OR)
**Dalsager** [24] Denmark	**Gestation** PFOS 8.1 (2.4–25.1) PFOA 1.7 (0.3–10.1) PFHxS 0.3 (0.0–1.0) PFNA 0.7 (0.2–3.6) PFDA 0.3 (0.1–1.7)		**1 year** (*n* = 346)	Fever (OR) Coughing (OR) Diarrhea (OR)
**Dalsager** [25] Denmark	**Gestation** PFOS 7.5 (2.4–25.1) PFOA 1.7 (0.3–10.1) PFHxS 0.4 (0.0–1.0) PFNA 0.6 (0.2–3.6) PFDA 0.3 (0.1–1.7)		**4 years** (*n* = 1472)	Hospitalization due to any infection (OR) Respiratory infections (OR) Gastrointestinal infections (OR)
**Fei** [26] Denmark	**Gestation** PFOS 35.3 (6.4–106.7)^c^ PFOA 5.6 (0 – 41.5)^c^		**9 years** (*n* = 1400)	Hospitalization due to any infection (OR)
**Goudarzi** [27] Japan	**Gestation** PFOS 4.9 (3.7–6.7) PFOA 2.0 (1.3–3.3) PFHxS 0.3 (0.2–0.4) PFNA 1.2 (0.9–1.6) PFDA 0.5 (0.4–0.7) PFUnDA 1.4 (1.0–1.9) PFDoDA 0.2 (0.1–0.2) PFTrDA 0.3 (0.2–0.4)		**4 years** (*n* = 1558)	Any infection (OR)
**Granum** [28] Norway	**Gestation** PFOS 5.6 (3.8–7.1) PFOA 1.1 (0.8–1.4) PFHxS 0.3 (0.3–0.4) PFNA 0.3 (0.2–0.4)		**3 years** (*n* = 49–93)	**Tetanus** (%D^d^) **Rubella** (%D) **Measles** (%D) **Hib** (%D) Cold^e^ (OR) Gastroenteritis^e^ (OR)
**Grandjean** [3] Faroe Islands	**Gestation** PFOS 27.3 (23.2–33.1) PFOA 3.2 (2.6–4.0) PFHxS 4.4 (2.2–8.4) PFNA 0.6 (0.5–0.8) PFDA 0.3 (0.2–0.4)	**5 years** PFOS 16.7 (13.5–21.1) PFOA 4.1 (3.3–5.0) PFHxS 0.6 (0.5–0.9) PFNA 1.0 (0.8–1.2) PFDA 0.3 (0.2–0.4)	**5 years** (*n* = 509^f^) **7 years** (*n* = 424)	**Tetanus** (%D) **Diphtheria** (%D)
**Grandjean** [20] Faroe Islands	**18 months**^g^ PFOS 18.5 (18.1–18.9) PFOA 7.1 (4.5–10.0) PFHxS 0.2 (0.1–0.4) PFNA 1.0 (0.6–1.5) PFDA 0.3 (0.2–0.4)	**5 years** PFOS 4.7 (3.5–6.3) PFOA 2.2 (1.8–2.8) PFHxS 0.3 (0.2–0.4) PFNA 1.0 (0.8–1.2) PFDA 0.3 (0.2–0.4)	**5 years** (*n* = 275–349)	**Tetanus** (%D) **Diphtheria** (%D)
**Grandjean** [21] Faroe Islands	**7 years** PFOS 15.3 (12.4–19.0) PFOA 4.4 (3.5–5.7) PFHxS 0.5 (0.4–0.7) PFNA 1.1 (0.9–1.5) PFDA 0.4 (0.2–0.6)	**13 years** PFOS 6.7 (5.2–8.5) PFOA 2.0 (1.6–2.5) PFHxS 0.4 (0.3–0.5) PFNA 0.7 (0.6–0.9) PFDA 0.3 (0.2–0.4)	**7 years** (*n* = 427) **13 years** (*n* = 505)	**Tetanus** (%D) **Diphtheria** (%D)
**Impinen** [29] Norway	**Gestation** PFOS 5.2 (4.0–6.6) PFOA 1.6 (1.2–2.1) PFHxS 0.2 (0.2–0.3) PFNA 0.2 (0.1–0.2) PFUnDA 0.1 (0.0–0.1) PFOSA 0.4 (0.2–0.5)		**2 years** (*n* = 640) **10 years** (*n* = 478)	Cold (OR) Atopic dermatitis (OR) Rhinitis (OR)
**Impinen** [30] Norway	**Gestation** PFOS 12.9 (9.9–16.6) PFOA 2.5 (1.8–3.3) PFHxS 0.7 (0.5–0.9) PFNA 0.5 (0.5–0.8) PFUnDA 0.2 (0.1–0.3) PFHpS (0.2 (0.1–0.2)		**3 years** **7 years** (*n* = 478–1207)	Cold^e^ (r/OR) Pheumonia^h^ (r/OR) Streptococcus (r/OR) Coughing^i^ (r/OR) Pseudocroup (r/OR) Otitis media (r/OR)^j^
**Mogensen** [31] Faroe Islands	**7 years** PFOS 15.5 (12.8–19.2) PFOA 4.4 (3.5–5.7) PFHxS 0.5 (0.4–0.7)		**7 years** (*n* = 443)	**Tetanus** (%D) **Diphtheria** (%D)
**Okada** [32] 2012 Japan	**Gestational** PFOS 5.2 (3.4–7.2) PFOA 1.3 (0.8–1.7)		**1.5 years** (*n* = 343)	Otitis media (OR)
**Stein** [33] USA	**14.5 years** PFOS 20.8 (19.1–22.7)^k^ PFOA 4.1 (3.8–4.5) PFHxS 2.4 (2.15–2.85) PFNA 0.8 (07–0.9)		**14.5 years** (*n* = 638–1190)	**Measles** (%D) **Mumps** (%D) **Rubella** (%D) Rhinitis (OR)
**Timmermann** [34] Guinea-Bissau	**4 months** PFOS 0.8 (0.5–1.0) PFOA 0.7 (0.5–0.9) PFHxS 0.1 (0.1–0.1) PFNA 0.2 (0.1–0.3) PFDA 0.2 (0.1–0.2) PFUnDA 0.1 (0.1–0.2)		**9 months** **2 years** (*N* = 91–236)	**Measles** (%D) Fever (r/OR) Coughing (r/OR) Diarrhea (r/OR)
**Zeng** [35] China	**Gestational** PFOA 1.2 (0.9–1.7) PFOS 3.2 (1.9–4.9) PFDA 0.1 (0.0–0.2) PFDoDA 0.1 (0.1–0.1) PFHxS 4.0 (2.3–5.4) PFUnDA 0.1 (0.0–0.3)		**0 months** **3 months** (*n* = 180–194)	**CA16** (%D) **EV71** (%D)
**Zhang** [22] USA	**7 years**^**l**^ PFOA 1.9 (NR^m^) PFOS 3.9 (NR) PFNA 0.8 (NR) PFHxS 0.8 (NR)		**7 years**^**l**^ (*n* = 517)	Cold^n^ (OR)

Bold = antibody

^a^As used in this report, ^b^odds ratio, ^c^range, ^d^percentage change in outcome per doubling of PFAS exposure, ^e^ever vs. never, ^f^pre-booster, ^g^gestational values are omitted as they were already published [3], ^h^bronchitis/pneumonia, ^i^other throat infection, ^j^report mentioned ear infection, ^k^geometric mean and 95% confidence interval, *r* correlation coefficient

PFAS and pediatric vaccine response, ^l^mean from published data, ^m^not reported, ^n^yes/no

Median (IQR) values for PFOS, the most prevalent of all PFAS, measured during gestation were generally low (values ranging from 1.2 to 12.9 ng/mL). Two studies [3, 26] reported higher in utero PFOS values with the compound being measured at 27.3 ng/mL and 35.3 ng/mL, respectively.

Childhood PFOS median values ranged from 0.8 to 18.5 ng/mL. Higher (20.8 ng/mL) childhood PFOS values were reported in one study with children aged 14 years [33].

PFOA values measured during gestation ranged from 1.1 to 5.6 ng/mL and those measured during childhood ranged from 0.7 to 7.1 ng/mL. The prevalence of the other PFAS measured during gestation was low with median values ranging from 0.2 to 4.4 ng/mL for PFHxS, 0.2 to 1.2 ng/mL for PFNA, 0.1 mg/mL to 0.5 ng/mL for PFDA, 0.1 to 1.4 ng/mL for PFUnDA, between 0.1 and 0.2 ng/mL for PFDoDA, and 0.3 ng/mL for PFTrDA, respectively. PFAS values measured during childhood ranged from 0.1 ng/mL to 2.4 ng/mL for PFHxS, 0.2 ng/mL to 1.1 ng/mL for PFNA, 0.3 mg/mL to 0.4ng/mL for PFDA and 0.1 ng/mL for PFUnDA, respectively.

Table 2 shows the results of the meta-analyses on % difference of tetanus, diphtheria, measles, mumps, haemophilus influenza type b (Hib), EV71, and CA16 antibody concentrations based on PFAS exposure measured during gestation (gestational) and during childhood.

**Table 2:** Adjusted percent change^a^ (95% confidence interval) in antibody titer with a doubling in per- and polyfluoroalkyl substances concentration measured during gestation and during childhood

PFAS	Antibody titer							
	Tetanus	Diphtheria	Measles	Mumps	Rubella	Haemophilus influenza B (Hib)	EV71^c^	CA16^c^
**PFOS** Gestational	2.1 (*k*^b^ = 4) (− 16.9, 21.2)	** − 24.3** (*k* = 3) **(− 39.2, − 9.4)**	** − 3.4** (*k* = 1) **(− 5.0, − 1.7)**		** − 5.4** (*k* = 1) **(− 9.2, − 1.4)**	− 10.5 (*k* = 1) (− 50.7, 62.5)	** − 23.6** (*k* = 1) **(− 33.9, − 11.8)**	** − 20.6** (*k* = 1) **(− 30.0, − 9.9)**
**PFOS** Childhood	− 3.2 (*k* = 7) (− 16.0, 9.7)	− 10.4 (*k* = 7) (− 25.3, 4.4)	− 9.4 (*k* = 3) (− 21.3, 2.4)	** − 7.4** (*k* = 1) **(− 12.8, − 1.7)**			** − 10.6** (*k* = 1) **(− 16.9, − 3.9)**	− 6.9 (*k* = 1) (− 13.9, 0.7)
**PFOA** Gestational	− 6.5 (*k* = 4) (− 19.2, 6.1)	** − 19.4** (*k* = 3) ** − 24.1, − 14.7)**	** − 8.6** (*k* = 1) **(− 15.1, − 2.1)**		** − 24.2** (*k* = 1) **(− 35.8, − 11.1)**	− 3.4 (*k* = 1) (− 93.1, 1236.1)	** − 18.7** (*k* = 1) **(− 28.6, − 7.4)**	
**PFOA** Childhood	** − 15** (*k* = 7) **(− 26.2, − 4.45)**	− 10.2 (*k* = 7) − 22.2, 1.7)	2.9 (*k* = 3) (− 2.4, 8.2)	− 6.0 (*κ* = 1) (− 12.4, 0.9)				
**PFHxS** Gestational	− 2.1 (*k* = 4) (− 10.0, 5.8)	** − 13.8** (*k* = 3) **(− 25.3, − 2.2)**	− 2.7 (*k* = 1) (− 10.8 5.3)		** − 23.4** (*k* = 1) **(− 36.7, − 7.3)**	− 28.3 (*k* = 1) (− 96.0, 1172)		
**PFHxS** Childhood	− 5.6 (*k* = 7) (− 15.0, 4.0)	− 3.5 (*k* = 7) (− 10.6, 3.6)	− 3.0 (*k* = 3) (− 6.6, 0.6)	− 2.6 (*k* = 1) (− 6.7, 1.7)				
**PFNA** Gestational	6.0 (*k* = 4) (− 6.5, 18.5)	− 5.6 (*k* = 3) (− 16.6, 5.3)	** − 31.7** (*k* = 1) **(− 49.0, − 14.3)**		** − 58.2** (*k* = 1) **(− 80.0, − 12.9)**			
**PFNA** Childhood	− 0.4 (*k* = 6) (− 14.1, 13.2)	− 6.0 (*k* = 6) (− 18.4, 6.4)	1.8 (*k* = 3) (− 3.6, 7.2)	− 2.7 (*k* = 1) (− 7.2, 2.0)				
**PFDA** Gestational	1.0 (*k* = 3) (− 15.9, 18.0)	− 8.5 (*k* = 3) (− 22.3, 5.2)						
**PFDA** Childhood	− 8.3 (*k* = 6) (− 11.6, 5.0)	− 7.0 (*k* = 6) (− 19.9 5.8)	− 10.2 (*k* = 2) − 41.0, 20.5)					
**PFUnDA** Childhood			− 11.5 (*k* = 2) (− 30.1, 7.1)					

^a^Effects are % per doubling of PFAS, ^b^number of comparisons (not number of studies), ^c^hand, foot, and mouth disease, bold: *p* < 0.05

A 2-fold increase in PFOS exposure (gestational) was associated with differences of + 2% − 24%, − 3%, − 5%, − 10%, − 24%, and − 21% for tetanus, diphtheria, measles, rubella, Hib, EV71, and CA16, respectively. The PFOS (childhood) exposure was associated with differences of − 3%, − 10%, − 9%, − 7%, − 11%, and − 7% for tetanus, diphtheria, measles, mumps, EV17, and CA16, respectively. A doubling of PFOA exposure (gestational) was associated with differences of − 7%, − 19%, − 9%, − 24%, − 3%, and − 19% for tetanus, diphtheria, measles, rubella, Hib, and EV71, respectively. The PFOA (childhood) exposure was associated with a difference of − 15%, − 10%, − 3%, and − 6% for tetanus, diphtheria, measles, and mumps, respectively. A doubling of PFHxS (gestational) exposure was associated with a difference of − 2%, − 14%, − 3%, − 23%, and − 28% for tetanus, diphtheria, measles, rubella, and Hib, respectively. The PFHxS (childhood) exposure was associated with differences of − 6%, − 4%, − 3%, and − 3% for tetanus, diphtheria, measles, and mumps, respectively. The PFNA (gestational) exposure was associated with differences of + 6%, − 6%, − 58%, and − 32% for tetanus, diphtheria, rubella, and measles, respectively. The PFNA (childhood) exposure was associated with differences of − 0%, − 6%, + 2%, and − 3% for tetanus, diphtheria, measles, and mumps, respectively. A doubling of PFDA (gestational) exposure was associated with differences of + 1% and − 9% for tetanus and diphtheria, respectively. The PFDA (childhood) exposure was associated with differences of − 8%, − 7%, and − 10% of tetanus, diphtheria, and measles, respectively. A doubling of PFUnDa (childhood) exposure was associated with a difference of − 11% for measles antibody concentration. In summary, a negative association between gestational PFOS, PFOA, PFHxS, and diphtheria (based on three comparisons from two individual studies) can be seen. Likewise, a negative association between childhood PFOA and tetanus (based on seven comparisons from four individual studies) is observed. With regard to the rest of the PFAS and antibody titers (Hib, EV17, CA16, rubella, measles, and mumps), there are significant negative associations; however, these are based only on one comparison.

### PFAS and childhood infections

Table 3 shows the results (odds ratios and 95% confidence intervals (CIs)) of the meta-analyses on the association between PFAS exposure and the prevalence of childhood infections. In adjusted models, the general pattern of the association between PFAS exposure and childhood infections appeared null. In most cases, odds ratios were below 1 for the development of a childhood infection. In essence, the results suggest that children with higher PFAS concentrations were less likely to develop a childhood infection. Nevertheless, children with higher PFOS exposures were more likely to develop a respiratory infection, any infection, and fever (OR, 1.17; 95% CI, 1.07–1.28; OR, 1.61; 95% CI, 1.18–2.20; and OR, 1.57; 95% CI, 1.21–2.03, respectively). Children with higher PFOA exposures were more likely to develop a respiratory infection and pseudocroup (OR, 1.21; 95% CI, 1.10–1.31; and OR, 1.22; 95% CI, 1.07–1.39, respectively). Children with higher PFHxS exposure were more likely to develop otitis media and pseudocroup (OR, 1.08; 95% CI, 1.03–1.13; and OR, 1.20; 95% CI, 1.11–1.30, respectively). In summary, a negative association between PFOS and respiratory infection (based on three comparisons from two individual studies) and fever (based on two comparisons from two individual studies) can be seen. A negative association between PFOA and respiratory infection (based on three comparisons from two individual studies) and pseudoucrop (based on one comparison from one study) was observed. A negative association between PFHxS and otitis media (based on three comparisons from two individual studies) and pseudocroup (based on one study) was observed. With regard to the rest of the PFAS and childhood infections, there were no associations observed.

**Table 3:** Adjusted associations (OR (95% confidence interval)) between per- and polyfluoroalkyl substances concentration and childhood infections

PFAS	Childhood infections											
	Throat infection^b^	Rhinitis^c^	Respiratory infection^d^	Otitis media	Gastrointestinal problems^e^	Dermatitis^f^	Hospitalization due to infection	Any infection^g^	Fever^h^	Cold	Pseudocroup	Chicken pox
**PFOS**	0.91 (*k*^a^ = 4) (0.84, 0.99)	1.03 (*k* = 3) (0.90, 1.18)	**1.17** (*k* = 4) **(1.07, 1.28)**	0.98 (*k* = 5) (0.85, 1.13)	1.03 (*k* = 4) (0.80, 1.32)	0.94 (*k* = 2) (0.57, 1.55)	1.13 (*k* = 2) (0.99, 1.30)	**1.61 (*k* = 1)** **(1.18, 2.20)**	**1.57** (*k* = 2) **(1.21, 2.03)**	0.92 (*k* = 3) (0.73, 1.16)	1.07 (*k* = 1) (0.96, 1.20)	1.10 (*k* = 1) (0.91, 1.32)
**PFOA**	1.05 (*k* = 4) (0.88, 1.26)	1.16 (*k* = 3) (0.91, 1.47)	**1.21** (*k* = 4) (**1.10, 1.31**)	1.02 (*k* = 3) (0.96, 1.09)	1.17 (*k* = 4) (0.60, 2.28)	1.15 (*k* = 2) (0.93, 1.42)	1.10 (*k* = 2) (0.77, 1.32)	1.11 (*k* = 1) 0.81, 1.26)	1.18 (*k* = 2) (0.88, 1.58)	0.96 (*k* = 3) (0.94, 0.99)	**1.22** (*k* = 1) (**1.07, 1.39**)	0.94 (*k* = 1) (0.81, 1.10)
**PFHxS**	1.05 (*k* = 4) (0.90, 1.23)	0.92 (*k* = 3) (0.82, 1.03)	1.08 (*k* = 4) (1.00, 1.18)	**1.08** (*k* = 3) **(1.03, 1.13)**	1.30 (*k* = 4) (0.82, 2.07)	1.05 (*k* = 2) (0.90, 1.23)	1.00 (*k* = 1) (0.89, 1.13)	0.96 (*k* = 1) (0.70, 1.31)	1.17 (*k* = 2) (0.88, 1.54)	1.19 (*k* = 3) (0.84 1.69)	**1.20** (*k* = 1) **(1.11, 1.30)**	0.87 (*k* = 1) (0.87, 1.00)
**PFNA**	1.07 (*k* = 4) (086, 1.32)	1.05 (*k* = 3) (0.81, 1.35)	1.03 (*k* = 4) (0.94, 1.12)	0.98 (*k* = 3) (0.89, 1.07)	0.91 (*k* = 4) (0.66, 1.26)	1.05 (*k* = 2) (0.88, 1.24)	1.04 (*k* = 1) (0.90, 1.20)	0.92 (*k* = 1) (0.67, 1.26)	1.14 (*k* = 2) (0.88, 1.46)	1.07 (*k* = 3) (0.82, 1.41)	0.98 (*k* = 1) (0.88, 1.10)	0.91 (*k* = 1) (0.76, 1.09)
**PFDA**	0.91 (*k* = 2) (0.72, 1.16)	0.82 (*k* = 1) (0.72, 0.94)	1.10 (*k* = 2) (0.96, 1.25)	0.92 (*k* = 1) (0.78 1.08)	0.85 (*k* = 3) (0.60, 1.21)		1.02 (*k* = 1) 0.89, 1.17)	0.80 (*k* = 1) (0.59, 1.09)	1.14 (*k* = 2) (0.88, 1.48)			1.01 (k = 1) (0.86, 1.19)
**PFDoDA**		0.96 (*k* = 1) (0.82, 1.12)	1.07 (*k* = 1) (0.90, 1.27)	0.94 (*k* = 1) 0.80, 1.10)								0.85 (*k* = 1) (0.72, 1.00)
**PFHpS**	1.00 (*k* = 2) (0.91, 1.11)		1.04 (*k* = 2) (0.79, 1.36)							0.99 (*k* = 1) (0.97, 1.01)	1.07 (*k* = 1) (0.99, 1.16)	
**PFOSA**		0.95 (*k* = 1) (0.79, 1.15)				1.11 (*k* = 2) (0.97, 1.27)						
**PFTrDA**		1.10 (*k* = 1) (0.96, 1.27)	1.05 (*k* = 1) (0.89, 1.24)	0.84 (*k* = 1) (0.72, 0.98)								1.02 (*k* = 1) (0.87, 1.20)
**PFUnDA**	1.05 (*k* = 3) (0.97, 1.15)	0.87 (*k* = 2) (0.78, 0.97)	1.03 (*k* = 3) (0.95, 1.12)	1.00 (*k* = 3) (0.85, 1.18)	0.91 (*k* = 1) (0.42, 1.96)	1.05 (*k* = 2) (0.88, 1.25)			0.71 (*k* = 1) (0.33, 1.53)	0.98 (*k* = 1) (0.96, 1.01)	0.86 (*k* = 1) (0.78, 0.95)	0.98 (*k* = 1) (0.85, 1.13)

^a^Number of comparisons across studies; ^b^throat category includes coughing and streptococcus infection; ^c^rhinitis category includes rhino-conjunctivitis; ^d^respiratory infection category includes bronchitis, pneumonia, and upper respiratory tract infection; ^e^gastrointestinal includes diarrhea and gastroenteritis; ^f^dermatitis category includes atopic dermatitis; ^g^any infection category includes at least one of the diseases otitis media, pneumonia, respiratory syncytial virus, and varicella; ^h^number of infections with fever

### Risk of bias

The overall risk of bias in the studies was considered low. However, some studies failed to adequately account for confounding variables or co-exposures, which may have introduced a certain level of bias.

### Certainty of evidence

Based on the percent difference in vaccine antibody response, there is *high certainty of confidence* in the results which suggest *no association* between:

- PFOS and measles, mumps (evidence from one study), and rubella (evidence from one study).
- PFOA and measles and Hib.
- PFHxS and tetanus and measles.
- PFNA and tetanus, mumps (evidence from one study), and diphtheria.
- PFDA and tetanus and diphtheria.

Based on the percent difference in vaccine antibody response, there is *low confidence of evidence* in the results which suggest *an association* between:

- PFOS and tetanus, diphtheria, Hib, EV71, and CA16.
- PFOA and tetanus, and diphtheria, and rubella (evidence from one study), and EV71 (evidence from one study).
- PFHxS and diphtheria, rubella (evidence from one study), and Hib (evidence from one study).
- PFNA and measles (evidence from one study) and rubella (evidence from one study).
- PFUnDA and measles.

Based on the likelihood of children developing an infectious disease during childhood there is *moderate certainty of evidence* in the results which suggest *an association* between:

- PFOS and respiratory infection and fever and any infection (evidence from one study).
- PFOA and respiratory infection and pseudocroup (evidence from one study).
- PFHxS and otitis media and pseudocroup (evidence from one study).

Based on the likelihood of children developing an infectious disease during childhood there is *high certainty of evidence* in the results which suggest *no association* between:

- PFOS and throat infection, rhinitis, otitis media, gastrointestinal problems, dermatitis, hospitalization due to infection, cold, pseudocroup, and chicken pox.
- PFOA and throat infection, rhinitis, otitis media, gastrointestinal problems, hospitalization due to infection, any infection, fever, cold, and chicken pox.
- PFHxS and throat infection, rhinitis, respiratory infection, gastrointestinal problems, dermatitis, hospitalization due to infection, any infection, fever, cold, and chicken pox.
- The individual compounds of PFNA, PFDA, PFDoDA, PFHpS, PFOSA, PFTRrDA, PFUnDA and throat infection, rhinitis, respiratory infection, otitis media, gastrointestinal problems, dermatitis, hospitalization due to infection, any infection, fever, cold, pseudocroup, and chicken pox.

## Discussion

Across the literature on PFAS exposure and child immune function, there is limited evidence of immunosuppressive effects related to reduced vaccine response. A detailed literature review and discussion of all studies included in this meta-analysis have been previously published by the authors of this meta-analysis [36].

The results of the combined data of all available studies conducted on the association between PFAS exposure during gestation and during childhood and antibody titers showed that any decrease in antibody response was evidently very small. Nevertheless, significant inverse associations were observed between anti-rubella concentrations measured at 3 years and PFOA, PFHxS, and PFNA serum concentrations. These effects were observed in only one study conducted with a small study population of children, where minor differences in the incidence of the various health outcomes may impact the statistical analyses and results.

Multiple exposure-health effects outcome comparisons were made in all studies included in the meta-analysis, and therefore some risk of bias cannot be ruled out, though this bias would be the same for all studies. 

Based on the summary estimates of the analyses, very small differences in antibody titers are seen across PFAS, independently of the level of exposure, and for the different vaccine antibody responses. Yet, it is not known whether the production of vaccine antibodies could be further impaired in case of exposures higher than those reported in the studies currently available. Moreover, only two studies (both conducted with the Faroese birth cohorts) reported associations with antibody titers falling below protective levels. Likewise, in cases where statistically relevant outcomes were observed (i.e., increased ORs for infectious diseases such as respiratory infections and fever), they were not consistent across studies and across outcome and were observed only for PFOS and PFOA.

A significant association was, however, observed between PFOA and PFHxS and pseudocroup, but the evidence is limited based on results from only one study.

The results of the meta-analyses should be considered in view of some limitations. Studies which reported only correlation data [37] were excluded as they could not be statistically combined with the rest of the studies. However, the summary statistics of these studies show no association between exposure and antibody response or infectious diseases.

Although some epidemiological studies suggested an inverse association between antibody response and PFAS exposure, the evidence is still weak due to a small number of studies, especially per vaccine type or per PFAS exposure, relatively high heterogeneity of study methodologies, variation of characteristics among the studies, design quality, and small effect sizes. 

In addition, results on the reduction of antibody levels are not consistent with an increased infection risk as a consequence of PFAS exposures. Therefore, the epidemiological evidence is not suggestive of an association. The extrapolation of the epidemiological data is further hampered by the fact that many of the associations were seen with high PFAS exposures in cohorts examined between 1997 and 2009 with PFAS exposures which are not observed anymore in contemporary populations. Although the releases of these compounds and especially PFOS, PFHxS, and PFOA have been considerably reduced since 2000, it should be noted that in certain populations average increases of PFAS concentrations have been observed [38].

Nevertheless, when we analyzed the influence of time on our data, there was no effect (data not shown). In addition, even though there were some significant increasing and decreasing exposure–response relationships found especially for PFOS and PFOA, a lack of comparability and consistency across the studies, as well as the small number of studies, obstructs the design of more informative statistical analyses. 

Moreover, one should be cautious in the interpretation of the results as a very large number of meta-analyses were performed and forest plots generated. Therefore, there may be some non-accounted publication bias or confounding arising from factors which have not been investigated in the studies.

## Conclusions

In general, the results of this meta-analysis on the epidemiologic evidence do not suggest a strong association between PFAS and immune conditions or infectious diseases in humans. Some associations were observed, but they lacked consistency across studies.

Future research should be designed to allow data collection from large health registries with clinically defined diseases investigated at different time points. The exposure windows, which have been analyzed in our meta-analyses, are a way to estimate the fluctuation of the PFAS concentrations throughout time and consider other environmental factors which could be unique for each exposure timing.

Prospective studies with real-world exposure data on PFAS and similar compounds such as methylmercury and PCBs and antibody responses on a variety of vaccinations are needed. Moreover, studies on the effects of various human toxicant exposures on vaccine responses and effectiveness have been proposed as a means to demonstrate that tests on immunotoxicity performed mainly with animals showing immune suppression can predict similar human responses [39, 40].

### Supplementary Information

Additional file 1: Tables S1–S19 and Figures S1–S7.

## Data Availability

The datasets used and/or analyzed during the current study are available from the corresponding author upon reasonable request.
