# Supplementary material for: Childhood PFAS exposure and immunotoxicity: a systematic review and meta-analysis of human studies
Source: Syst Rev. 2024 Jul 9;13:176. doi: 10.1186/s13643-024-02596-z (PMC11232141; doi:10.1186/s13643-024-02596-z)
Supplement: Supplementary file 1 — Additional file 1: Tables S1–S19 and Figures S1–S7. [file 13643_2024_2596_MOESM1_ESM.docx]

Supplementary Information

Table S1 Risk of Bias Heatmap for studies included in meta-analysis


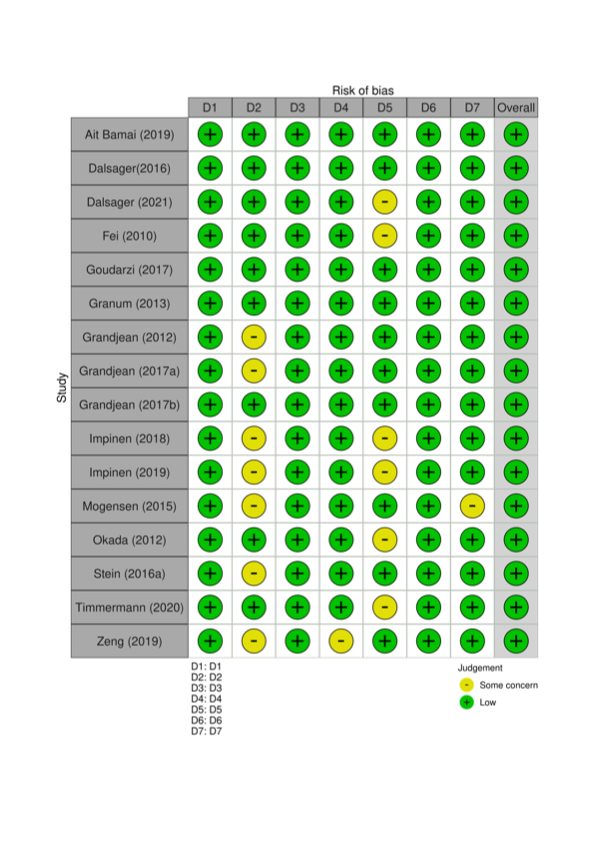


| D1. Did selection of study participants result in appropriate comparison groups? |
| --- |
| D2. Did the study design or analysis account for important confounding and modifying variables? |
| D3. Were outcome data complete without attrition or exclusion from analysis? |
| D4. Can we be confident in the exposure characterization? |
| D5. Can we be confident in the outcome assessment? |
| D6. Were all measured outcomes reported? |
| D7. Were there no other potential threats to internal validity? |

Table S2 Confidence ratings by key features


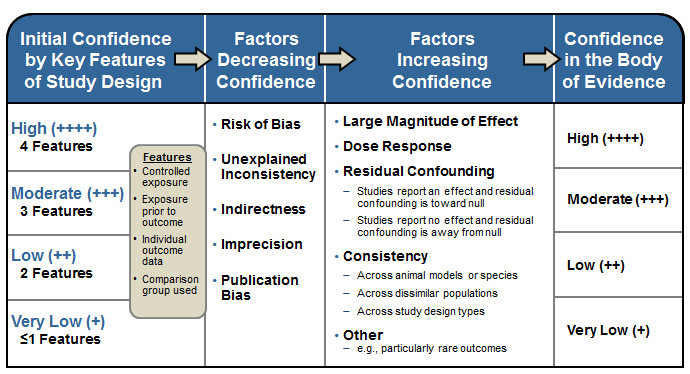


Graph presented in: National Toxicology Program, Handbook for Conducting a Literature-Based Health Assessment Using OHAT Approach for Systematic Review and Evidence Integration. 2015, National Institute of Environmental Health Sciences.U.S.A.

Table S3

| **High confidence** (++++) in the association between exposure to the substance and the  outcome. The true effect is highly likely to be reflected in the apparent relationship. |
| --- |
| **Moderate Confidence (+++)** in the association between exposure to the substance and the outcome. The true effect may be reflected in the apparent relationship. |
| **Low Confidence (++)** in the association between exposure to the substance and the outcome. The true effect may be different from the apparent relationship. |
| **Very Low Confidence (+)** in the association between exposure to the substance and the outcome. The true effect is highly likely to be different from the apparent relationship. |

As presented in: National Toxicology Program, Handbook for Conducting a Literature-Based Health Assessment Using OHAT Approach for Systematic Review and Evidence Integration. 2015, National Institute of Environmental Health Sciences. United States.

Table S4 GRADE assessment for the association between exposure to PFOS vaccination antibody levels


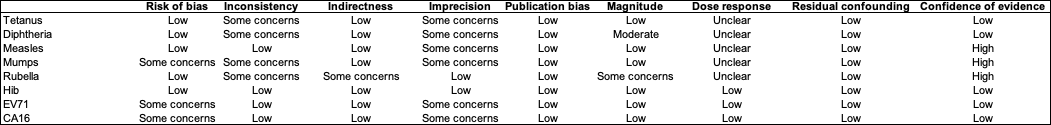


Table S5 GRADE assessment for the association between exposure to PFOS and childhood infections


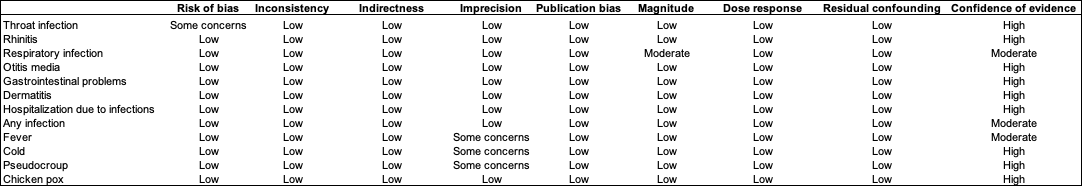


Table S6 GRADE assessment for the association between exposure to PFOA vaccination antibody levels


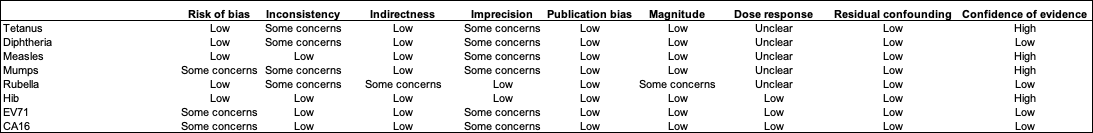


Table S7 GRADE assessment for the association between exposure to PFOA and childhood infections


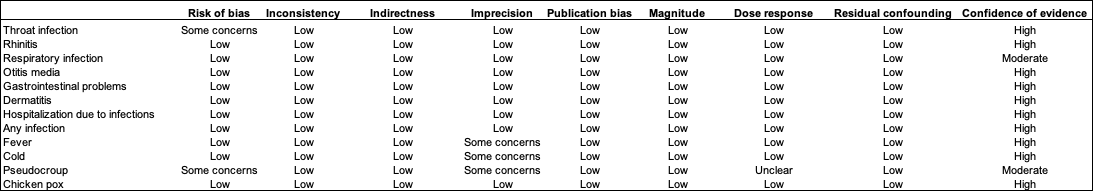


Table S8 GRADE assessment for the association between exposure to PFHxS vaccination antibody levels


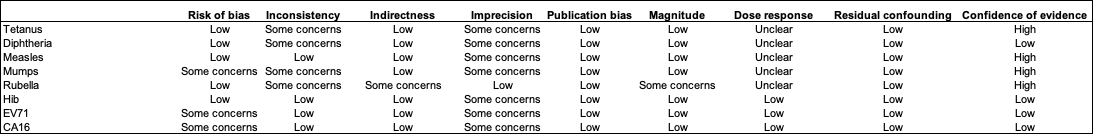


Table S9 GRADE assessment for the association between exposure to PFHxS and childhood infections


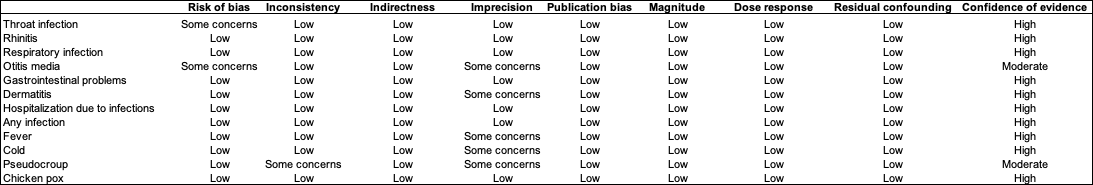


Table S10 GRADE assessment for the association between exposure to PFNA vaccination antibody levels


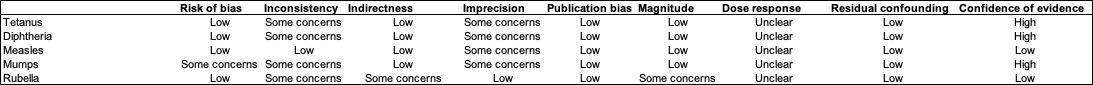


Table S11 GRADE assessment for the association between exposure to PFNA and childhood infections


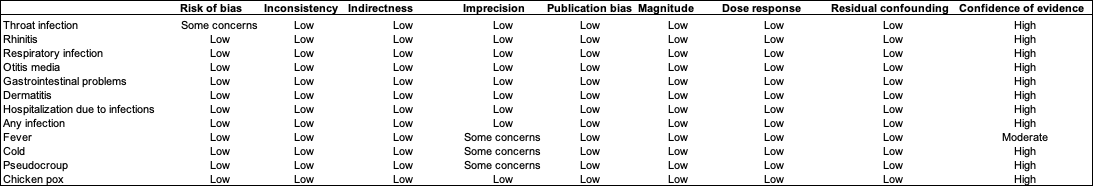


Table S12 GRADE assessment for the association between exposure to PFDA vaccination antibody levels


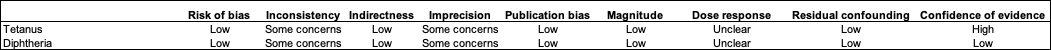


Table S13 GRADE assessment for the association between exposure to PFDA and childhood infections


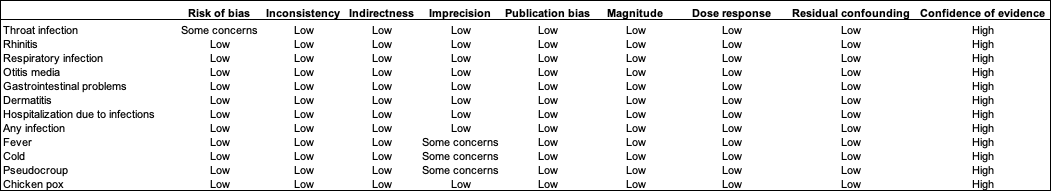


Table S14 GRADE assessment for the association between exposure to PFDoDA and childhood infections


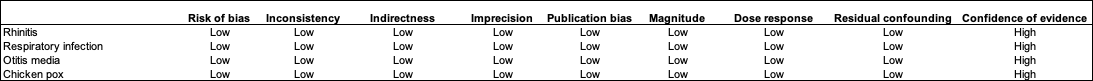


Table S15 GRADE assessment for the association between exposure to PFHpS and childhood infections


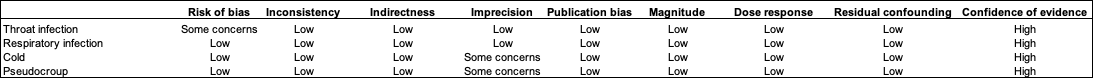


Table S16 GRADE assessment for the association between exposure to PFOSA and childhood infections


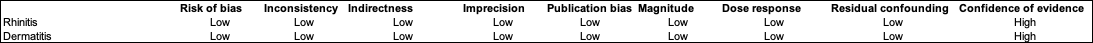


Table S17 GRADE assessment for the association between exposure to PFTrDA and childhood infections


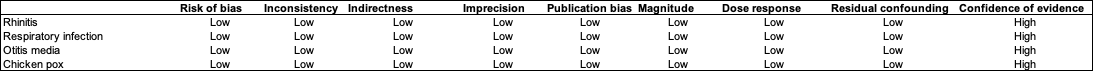


Table S18 GRADE assessment for the association between exposure to PFUnDA and vaccination antibody levels


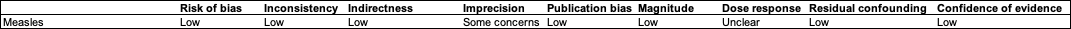


Table S19 GRADE assessment for the association between exposure to PFUnDA and childhood infections


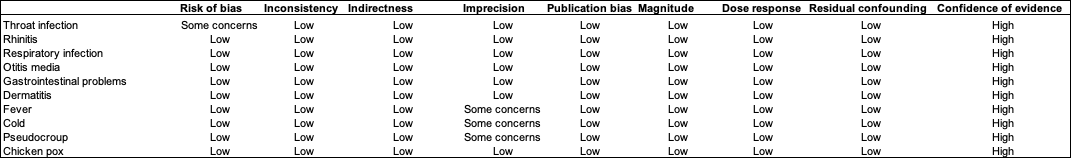


Figure S1 Adjusted forest plots for the association between PFOS exposure, vaccine antibody levels and infections in children. (1) PFOS exposure and tetanus; (2) PFOS exposure and diphtheria; (3) PFOS exposure and measles; (4) PFOS exposure and infections

| (1) | (2) |
| --- | --- |
|  |  |

| (3) |
| --- |
|  |

(4)

Figure S2 Adjusted forest plots for the association between PFOA exposure, vaccine antibody levels and infections in children. (5) PFOA exposure and tetanus; (6) PFOA exposure and diphtheria; (7) PFOA exposure and measles; (8) PFOA and infections

| (5) | (6) |
| --- | --- |
|  |  |
| (7) |  |
|  |  |

| (8) |  |
| --- | --- |
|  |  |

Figure S3 Adjusted forest plots for the association between PFHxS exposure, vaccine antibody levels and infections in children. (9) PFHxS exposure and tetanus; (10) PFHxS exposure and diphtheria; (11) PFHxS exposure and measles; (12) PFHxS and infections

| (9) | (10) |
| --- | --- |
|  |  |
|  |  |
| (11)    (12)   |  |

Figure S4 Adjusted forest plots for the association between PFNA exposure, vaccine antibody levels and infections in children. (13) PFNA exposure and tetanus; (14) PFNA exposure and diphtheria; (15) PFNA exposure and measles; (16) PFNA and infections

| (13) | (14) |
| --- | --- |
|  |  |
| \| (15) \| \| --- \| \|  \|   (16)   |  |

Figure S5 Adjusted forest plots for the association between PFDA exposure, vaccine antibody levels and infections in children. (17) PFDA exposure and tetanus; (18) PFDA exposure and diphtheria; (19) PFDA exposure and measles; (20) PFDA and infections

| (17) | (18) |
| --- | --- |
|  |  |
| (19) |  |
|  |  |

(20)

Figure S6 Adjusted forest plots for the association between PFUnDA exposure, vaccine antibody levels and infections in children. (21)

PFUnDA exposure and measles; (22) PFUnDA exposure and infections

(21) (22)

Figure S7 Adjusted forest plots for the association between PFHpS exposure and infections in children.

(23)
